# COVID-19 mRNA vaccination is associated with IgA nephropathy: an analysis of the Japanese adverse drug event report database

**DOI:** 10.3389/jpps.2023.11453

**Published:** 2023-06-30

**Authors:** Hiroka Nakao, Takenao Koseki, Koki Kato, Shigeki Yamada, Naotake Tsuboi, Kazuo Takahashi, Tomohiro Mizuno

**Affiliations:** ^1^ Department of Biomedical Molecular Sciences, School of Medicine, Fujita Health University, Toyoake, Japan; ^2^ Department of Pharmacotherapeutics and Informatics, School of Medicine, Fujita Health University, Toyoake, Japan; ^3^ Department of Nephrology, School of Medicine, Fujita Health University, Toyoake, Japan

**Keywords:** COVID-19, mRNA vaccine, IgA nephropathy, Japanese adverse drug event report, reporting odds ratio

## Abstract

**Purpose:** Coronavirus disease 2019 (COVID-19) mRNA vaccines are used worldwide to prevent severe symptoms of severe acute respiratory syndrome coronavirus 2 (SARS-CoV-2) infection. IgA nephropathy (IgAN) is the most common form of glomerular injury after COVID-19 vaccination; however, because of the low frequency of such events, only a few reports have been published. A large pharmacovigilance database of real-world spontaneous adverse event (AE) reports is essential for evaluating the drug-associated safety signals regarding rare AEs. Herein, we aimed to investigate the frequency of IgAN after the COVID-19 vaccination, using the Japanese Adverse Drug Event Report (JADER) database.

**Methods:** Data on drug-associated AEs reported between April 2004 and May 2022 were obtained from the JADER database on the Pharmaceuticals and Medical Devices Agency website. To evaluate the safety signals for the targeted AEs, reporting odds ratios (RORs), information components (ICs), and their 95% confidence intervals (CIs) were calculated using two-by-two contingency tables.

**Results:** A total of 697,885 cases were included in the analysis. Safety signals were detected for IgAN (ROR: 6.49, 95% CI: 4.38–9.61; IC: 2.27, 95% CI: 1.70–2.83). Of 30 cases for IgAN associated with COVID-19 mRNA vaccines, 16 had information available on time to onset. Of the 16 cases, 11 occurred ≤2 days after vaccination, and two occurred >28 days after vaccination.

**Conclusion:** These results suggest that, compared with other drugs, COVID-19 vaccination is associated with a higher frequency of IgAN. Monitoring of gross hematuria following COVID-19 vaccination should be needed.

## Introduction

The coronavirus disease 2019 (COVID-19) pandemic has caused severe health and economic damage. To prevent the symptoms of severe acute respiratory syndrome coronavirus 2 (SARS-CoV-2) infection, COVID-19 mRNA vaccines are being used worldwide. The mRNA is delivered by lipid nanoparticles into the cells and translated to the target protein which promotes a robust immune response [1, 2]. mRNA vaccines are associated with a risk of glomerular nephropathy [3, 4], and relapse of minimal change disease has been reported after COVID-19 vaccination [3, 5, 6].

IgA nephropathy (IgAN) is a type of glomerulonephritis characterized by the deposition of IgA1-containing immune complexes. Previous case reports suggest that COVID-19 vaccination is associated with a relapse of IgAN [7, 8]. These patients presented with gross hematuria within 24 h after receiving the second dose of a COVID-19 mRNA vaccine, and the hematuria resolved within 3–5 days. Although these cases improved without immunosuppressive therapy, a case of new-onset IgAN with severe crescentic glomerulonephritis has been reported after the second vaccination [9]. This case showed mild mesangial expansion, endocapillary hypercellularity, and IgA positivity in granular mesangial cells. Immunosuppressive therapy was initiated after the patient was diagnosed with IgA vasculitis. Kidney function and proteinuria improved after administering pulse intravenous methylprednisolone. Ma and Xu [10] reviewed nine cases of new-onset IgAN after the first vaccination. The median time to onset was 10 days and immunotherapy was needed in seven cases. Thirty-eight cases of IgAN were reported after the second vaccination, including 22 new cases, with a median time to onset of 2 days. IgAN is thought to be the most common glomerular injury after COVID-19 vaccination; however, there are few published reports. In addition, information regarding time to onset (TTO) and outcomes is limited because the frequency of IgAN is lower than that of other adverse events (AEs) following vaccination.

A large pharmacovigilance database of real-world spontaneously reported AEs is essential for evaluating the drug-associated safety signals regarding rare AEs. The Japanese Adverse Drug Event Report (JADER) is a nationwide database of spontaneously reported AEs developed by the Pharmaceuticals and Medical Devices Agency (PMDA), the pharmaceutical regulatory authority in Japan. The calculation of reporting odds ratios (RORs) and information components (ICs) from the JADER database are recognized as indicators for the investigation of drug-associated safety signals [11–14]. Herein, we aimed to investigate the frequency of IgAN after COVID-19 mRNA vaccination by calculating RORs and ICs using the JADER database.

## Materials and methods

### Data source and data processing

**FIGURE 1:**
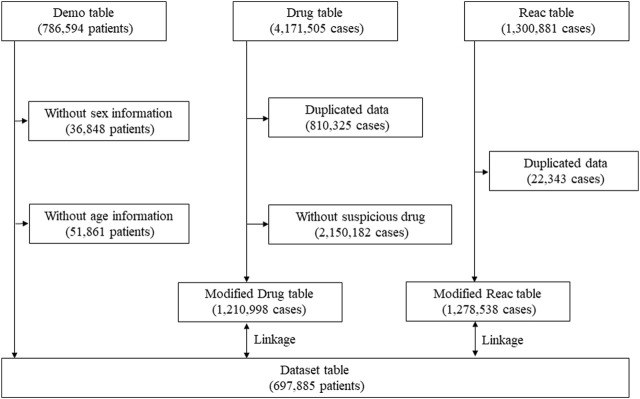
Flow diagram of the study.

Data extraction was conducted according to previous reports [12, 13, 15]. Briefly, data were obtained from the JADER database between April 2004 and May 2022. Because COVID-19 vaccine was introduced in our country in February 2021 (and in January 2022 for children aged 5–11 years), the database covers the report regarding the abovementioned population. All data in this study were obtained from the PMDA website (https://www.pmda.go.jp/english/index.html). The JADER dataset consists of demographic information “demo,” drug information “drug,” and adverse reaction information “reac” tables. Cases lacking information on sex or age in the “demo” table, and those with duplicate records in the “drug” and “reac” tables were excluded. The medication was classified into three categories: “suspected drug,” “concomitant drug,” and “interaction drug” depending on the contribution to the AE. The cases in the “suspected drug” category were evaluated in this study. To evaluate the safety signals for IgAN, nephritis, and lupus nephritis in patients after COVID-19 mRNA vaccination, COVID-19 mRNA vaccine (recorded as “Coronavirus Modified Uridine RNA Vaccine [SARS-CoV-2]”) was selected from the “drug” table. The “demo” table was linked to the “drug” and “reac” tables using the case identification number (Figure 1).

### Definition of IgA nephropathy, nephritis, and lupus nephritis

In the JADER database, the medical conditions are defined according to the Medical Dictionary for Regulatory Activities (MedDRA) list of preferred terms (PTs). The AEs in the “reac” table are defined based on the PTs in MedDRA version 25.1. In this study, IgAN, nephritis, and lupus nephritis were selected as the targeted AEs from 37 PTs by two nephrologists (Table 1).

**TABLE 1:** Definition of IgA nephropathy, nephritis, and lupus nephritis.

PT code	PT name
IgA nephropathy	
10021263	IgA nephropathy
Nephritis	
10018364	Glomerulonephritis
10018366	Glomerulonephritis acute
10018367	Glomerulonephritis chronic
10018370	Glomerulonephritis membranoproliferative
10018376	Glomerulonephritis proliferative
10018378	Glomerulonephritis rapidly progressive
10029120	Nephritis allergic
10029164	Nephrotic syndrome
10065673	Nephritic syndrome
10066453	Mesangioproliferative glomerulonephritis
10067757	Focal segmental glomerulosclerosis
10073016	Chronic autoimmune glomerulonephritis
10075626	Paraneoplastic nephrotic syndrome
10076749	Paraneoplastic glomerulonephritis
10029117	Nephritis
10048302	Tubulointerstitial nephritis
10069034	Tubulointerstitial nephritis and uveitis syndrome
10077087	Autoimmune nephritis
10083070	Immune-mediated nephritis
10020586	Hypercalcaemic nephropathy
10029151	Nephropathy
10037111	Pseudo-Bartter syndrome
10038457	Renal glycosuria
10038535	Renal tubular acidosis
10038536	Renal tubular atrophy
10038537	Renal tubular disorder
10050335	Renal tubular dysfunction
10050839	Bartter’s syndrome
10051920	Glomerulonephropathy
10052313	Liddle’s syndrome
10052607	Fanconi syndrome acquired
10054832	Diffuse mesangial sclerosis
10061989	Glomerulosclerosis
10062906	Gitelman’s syndrome
10075849	Potassium wasting nephropathy
10080593	Pseudohypoaldosteronism
10083522	Immune-mediated renal disorder
Lupus nephritis	
10025140	Lupus nephritis

PT, preferred term.

### Signal detection

**FIGURE 2:**
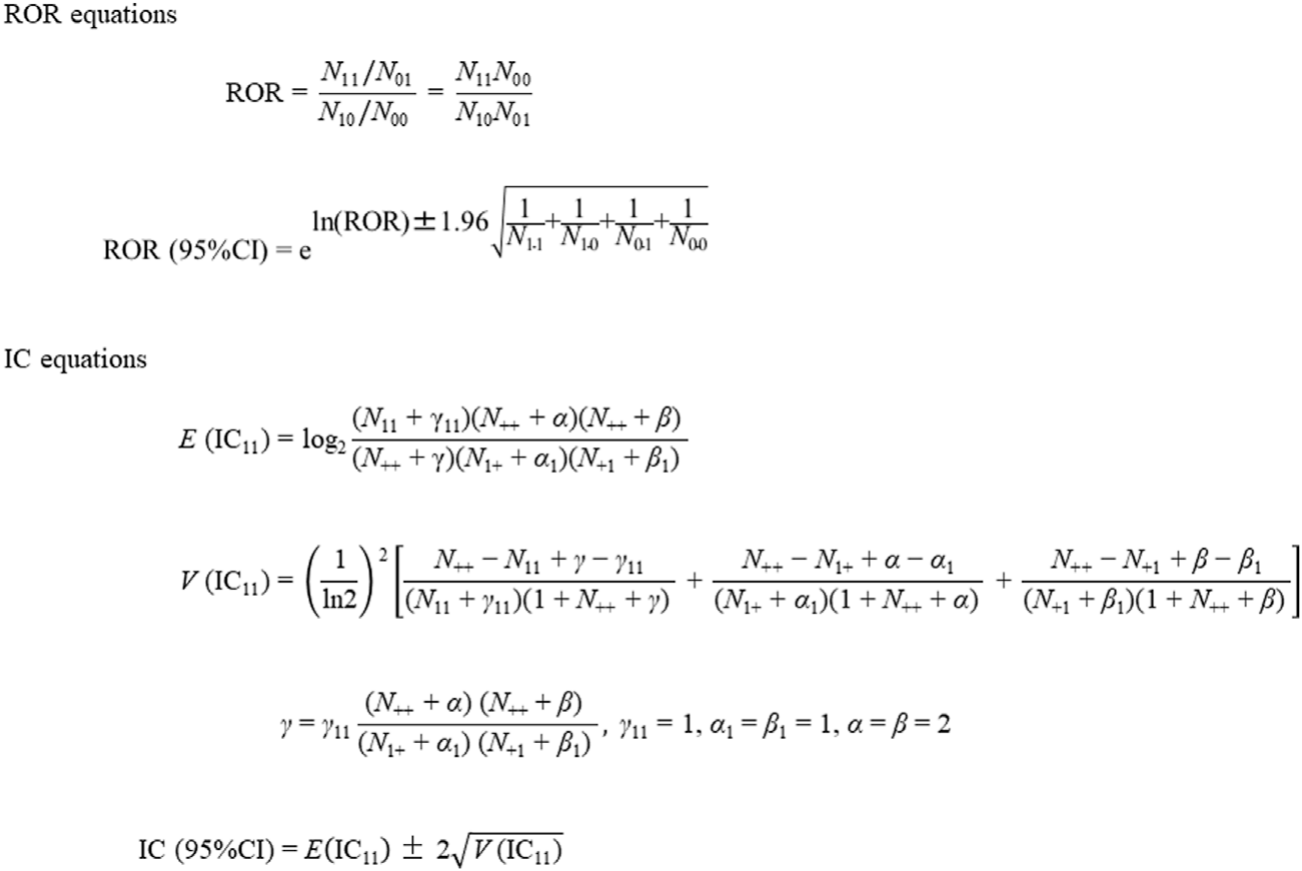
Reporting odds ratio and information component equations.

To evaluate the safety signals for targeted AEs, RORs, ICs, and their 95% confidence intervals (CIs) were calculated using two-by-two contingency tables (Table 2) and equations (Figure 2), as described previously [11, 12]. Briefly, RORs and ICs were calculated from a cross-tabulation table based on the number of cases of IgAN associated with COVID-19 mRNA vaccination using the equations shown in Figure 2. We calculated the RORs and ICs using Microsoft 365 Excel (Microsoft Corporation, Redmond, WA, United States). The safety signals for IgAN, nephritis, and lupus nephritis were positive if the lower limit of the 95% CI of the ROR was >1 and that of the IC was >0 [11, 12].

**TABLE 2:** Two-by-two contingency table.

	Target AEs	Other AEs	Total
Target drugs	*N*_*11*_	*N*_*10*_	*N*_*1+*_
Other drugs	*N*_*01*_	*N*_*00*_	*N*_*0+*_
Total	*N*_*+1*_	*N*_*+0*_	*N*_*++*_

AEs, Adverse events; N, number of cases.

### Time to onset assessment

TTO was calculated from the date of the first COVID-19 mRNA vaccination in the “drug” table to the date of the first occurrence of the IgAN recorded in the “reac” table. Patients with missing values or those without data were classified as unknown.

### Ethics approval and consent to participate

As this study used an open-access database, ethics approval and consent to participate were not required.

## Results

### Patient characteristics

A total of 697,885 cases were included in the analysis. Their sex and age characteristics are shown in Table 3. The number of all AEs associated with COVID-19 mRNA vaccination was 21,455. The frequency of drug-induced nephritis was lower in females than that in males. The frequency of drug-induced IgAN was similar between males and females, while that of drug-induced lupus nephritis was higher in females than that in males. Although the number of AEs reported for all drugs was similar between females and males, the number of reported AEs associated with COVID-19 mRNA vaccine was higher in females than that in males. The number of all AEs reported for the COVID-19 mRNA vaccine was lower in the under 10s than that in the other age groups. The number of cases of IgAN reported as AEs associated with COVID-19 mRNA vaccination was higher in females and younger age groups (10–49 years) than that in males and older age groups (≥50 years), respectively. The number of cases of nephritis and lupus nephritis were 98 and 0, respectively. The occurrence of nephritis reported as AEs associated with COVID-19 mRNA vaccination did not differ by sex or age.

**TABLE 3:** Sex- and age-specific case population for IgA nephropathy, nephritis, and lupus nephritis.

	Sex		Age (years)											Total
	Male	Female	<10	10s	20s	30s	40s	50s	60s	70s	80s	90s	100s	
All drugs														
All AEs	356,723 (51.11)	341,162 (48.89)	25,152 (3.60)	20,318 (2.91)	25,503 (3.65)	40,245 (5.77)	56,683 (8.12)	88,673 (12.71)	154,485 (22.14)	180,378 (25.85)	92,752 (25.85)	13,419 (1.92)	277 (0.04)	697,885
IgA nephropathy	90 (51.14)	86 (48.86)	6 (3.41)	16 (9.09)	25 (14.20)	20 (11.36)	27 (15.34)	26 (14.77)	31 (17.61)	21 (11.93)	4 (2.27)	0 (0)	0 (0)	176
Nephritis	3,077 (57.41)	2,283 (42.59)	303 (5.65)	362 (6.75)	192 (3.58)	313 (5.84)	481 (8.97)	702 (13.10)	1,204 (22.46)	1,307 (24.38)	464 (8.66)	32 (0.60)	0 (0)	5,360
Lupus nephritis	14 (22.58)	48 (77.42)	2 (3.23)	3 (4.84)	8 (12.90)	10 (16.13)	12 (19.35)	5 (8.06)	16 (25.81)	3 (4.84)	3 (4.84)	0 (0)	0 (0)	62
COVID-19 mRNA vaccines														
All AEs	7,386 (34.43)	14,069 (65.57)	51 (0.24)	1,353 (6.31)	2,916 (13.59)	2,893 (13.48)	3,594 (16.75)	2,896 (13.50)	2,156 (10.05)	2,577 (12.01)	2,221 (10.35)	762 (3.55)	36 (0.17)	21,455
IgA nephropathy	9 (30.00)	21 (70.00)	0 (0)	9 (30.00)	6 (20.00)	3 (10.00)	7 (23.33)	2 (6.67)	1 (3.33)	2 (6.67)	0 (0)	0 (0)	0 (0)	30
Nephritis	49 (50.00)	49 (50.00)	0 (0)	14 (14.29)	11 (11.22)	7 (7.14)	17 (17.35)	5 (5.10)	18 (18.37)	13 (13.27)	11 (11.22)	2 (2.04)	0 (0)	98
Lupus nephritis	0	0	0	0	0	0	0	0	0	0	0	0	0	0

Cases (%). AEs, adverse events.

### Safety signals of IgA nephropathy and nephritis

The RORs and CIs of IgAN and nephritis reported as AEs of COVID-19 mRNA vaccines are shown in Table 4. Safety signals were detected for IgAN (ROR, 6.49 [95% CI 4.38–9.61] and IC, 2.27 [95% CI 1.70–2.83]), but not for nephritis (ROR, 0.59 [95% CI 0.48–0.72] and IC, −0.74 [95% CI −1.04 to −0.45]).

**TABLE 4:** Reporting odds ratios and information components of COVID-19 mRNA vaccines for IgA nephropathy and nephritis.

	ROR [95% CI]	IC [95% CI]
IgA nephropathy	6.49 [4.38–9.61]	2.27 [1.70–2.83]
Nephritis	0.59 [0.48–0.72]	−0.74 [−1.04 to −0.45]

CI, confidence intervals; IC, information component; ROR, reporting odds ratio.

### Time to onset and outcomes of IgA nephropathy

In 30 cases of IgAN associated with COVID-19 mRNA vaccine, 16 cases had information available on TTO. Among the 16 cases, 11 cases had a TTO of <2 days and 2 cases had a TTO of >28 days after the vaccination (Table 5). In addition, information on outcomes was obtained on 19 IgAN cases associated with COVID-19 mRNA vaccines. Among the 19 patients, 8 patients were in recovery or remission, and 11 patients were not in recovery (Table 5).

**TABLE 5:** Time-to-onset and outcomes of occurrence of IgA nephropathy in cases with COVID-19 mRNA vaccines.

Time-to-onset	N	Outcomes	N
0 day (the same day)	2	Recovery	2
1 day	5	Remission	6
2 days	4	No recovery	11
3–28 days	3	After-effect	0
28< days	2	Death	0
Unknown	14	Unknown	11

Time-to-onset is the period from the most recent vaccination day to the first occurrence day of IgA nephropathy. N, number of cases.

## Discussion

The results of this study suggest that COVID-19 vaccination increases the frequency of IgAN more than that of other drugs. In addition, females and young individuals show the tendency of an increased frequency of IgAN associated with COVID-19 vaccination compared to that of males and older individuals. Because the frequency of drug-induced nephritis was higher in males than in females, there might be different mechanisms in nephritis associated with COVID-19 vaccination and that associated with other drugs. Most of drug-induced nephritis cases are characterized by tubular necrosis, interstitial nephritis, and tubular obstruction [16]. The causes of this nephritis are administration of antibiotics, anticancer agents, and non-steroidal anti-inflammatory agents, and the frequency is higher in males than in females [17]. Conversely, the impact of sex difference is small in some forms of glomerulonephritis including IgAN [18]. Although the JADER database does not include information regarding renal biopsy, most of nephritis cases associated with COVID-19 vaccination might be glomerulonephritis.

A questionnaire survey conducted in Japan [19] contained questions on the frequency and clinical features of gross hematuria after COVID-19 vaccination. The survey included 27 cases with gross hematuria after COVID-19 vaccination. Most of the patients with gross hematuria were aged 20–29 years, and 81.5% of the cases were in females. Our results are similar to those of the mentioned survey; however, the survey had selection bias because the participants included council members of the Japanese Society of Nephrology (382 facilities), and the participants tended to be from large hospitals. Furthermore, the association between the onset of gross hematuria and COVID-19 vaccination was not evaluated using statistical methods. To avoid selection bias in this study, we used a nationwide database of spontaneous reports of AEs. Although the JADER database might include the same cases included in the survey [19], the database contained 30 reported cases of IgAN. Therefore, our analysis included some cases that were not included in the survey. All patients diagnosed with IgAN developed gross hematuria within 14 days after COVID-19 vaccination in the abovementioned survey, whereas among the cases of IgAN in the JADER database, two occurred more than 28 days after vaccination. Our results suggest that careful monitoring for gross hematuria is needed for 1–2 months after COVID-19 vaccination.

Over 70% of cases with gross hematuria were diagnosed with IgAN in the previous survey [19]. In our study, the number of cases with recovery and remission was 8, whereas 11 patients did not recover. Because we could not ascertain from the data in the JADER database whether the cases of IgAN were relapses or newly diagnosed, the clinical features of the cases are unclear. In most previous case reports, the TTO of IgAN was <7 days [20]; therefore, cases that developed within 7 days might be a relapse of IgAN triggered by COVID-19 vaccination. The frequency of “nephritis” was not increased and no cases of lupus nephritis were detected after COVID-19 vaccination. Because the JADER database is a database of spontaneous reports of AE, many cases are not classified as typical nephritis. However, the frequency of IgAN was increased after COVID-19 vaccination. It is speculated that the mechanism of IgAN after COVID-19 vaccination is a multi-hit process caused by a combination of pre-existing IgA_1_ bearing galactose-deficient O-glycans and an increase in spike antigen-specific IgA levels after COVID-19 vaccination [10, 21]. Based on this hypothesis, the incidence of IgAN relapse is likely to increase after COVID-19 vaccination.

Mucosal IgA is produced in the early stage and shows more potent neutralizing capacity than that of systemic IgG in immune responses against SARS-CoV-2 infection [22]. Therefore, mucosal immunity is the main target for development of new COVID-19 vaccines [23–25]. Since the development of vaccines targeting mucosal immunity continues, our results could contribute to the detection and monitoring of IgAN following COVID-19 vaccination. The incidence rate of IgAN in Asian countries is higher than that in European countries. The results of this study could influence decision-making regarding COVID-19 vaccination and make clinicians aware of the need to screen patients with gross hematuria for IgAN.

Our study has several limitations. First, a high incidence of systemic AEs has been reported for the COVID-19 mRNA vaccine, and the incidence of AEs is higher with the second dose than that with the first dose [26, 27]; however, in this study, it was unclear whether the cases had an onset after the first or the second dose of the vaccine. Therefore, our study could not clarify whether the vaccine booster relationship and dosing gap affect the frequency of IgAN. Second, the generic drug name “Coronavirus Modified Uridine RNA Vaccine (SARS-CoV-2)” defined as COVID-19 mRNA vaccine does not distinguish between Pfizer-BioNTech and Moderna vaccines. The further studies with data recorded all types of vaccination should be needed to support our results. Third, the association between the IgAN and COVID-19 vaccination was based on the diagnosis of physicians who reported cases to JADER; however spontaneous reporting systems such as JADER are passive reporting systems. There are subject to many biases, including under-reporting, over-reporting, and confounding by comorbidities. To avoid these biases, further studies should compare the incidence rate of IgAN in populations of people who have undergone COVID-19 mRNA vaccination with that of unvaccinated people. In future studies, the analyses should be adjusted for comorbidities and other medications which affect the immune system to minimize the effect of confounding by these factors. In addition, analyses should be stratified by the vaccine type and the number of doses of vaccine received.

## Conclusion

The analyses revealed that COVID-19 vaccination is associated with an increased incidence of IgAN. Monitoring of gross hematuria following COVID-19 vaccination is needed. In addition, further studies should be conducted to confirm our findings.

## Novelty of the work

This study investigated the frequency of IgAN after the COVID-19 vaccination using the large nationwide Japanese Adverse Drug Event Report (JADER) database. We revealed the association between the onset of IgAN and COVID-19 vaccination by using statistical methods.

## Data Availability

The datasets presented in this article are not readily available because The Pharmaceuticals and Medical Devices Agency, which owns this data, does not permit the direct sharing of the data. JADER is a freely available public database. Requests to access the datasets should be directed to https://www.pmda.go.jp/english/index.html.

## Abbreviations

AE, adverse event; CI, confidence interval; COVID-19, coronavirus disease 2019; IC, information component; IgAN, IgA nephropathy; JADER, Japanese adverse drug event report; MedDRA, medical dictionary for regulatory activities; PMDA, pharmaceuticals and medical devices agency; PT, preferred term; ROR, reporting odds ratio; SARS-CoV-2, severe acute respiratory syndrome coronavirus 2; TTO, time to onset.
